# A State-of-the-Art Review on Soil Reinforcement Technology Using Natural Plant Fiber Materials: Past Findings, Present Trends and Future Directions

**DOI:** 10.3390/ma11040553

**Published:** 2018-04-04

**Authors:** Sivakumar Gowthaman, Kazunori Nakashima, Satoru Kawasaki

**Affiliations:** 1 Graduate School of Engineering, Hokkaido University, Sapporo 060-8628, Japan; 2 Faculty of Engineering, Hokkaido University, Sapporo 060-8628, Japan; nakashima@geo-er.eng.hokudai.ac.jp (K.N.); kawasaki@geo-er.eng.hokudai.ac.jp (S.K.)

**Keywords:** natural fibers, synthetic material, biochemical properties, sustainable geotechnics, oriented distributed fiber-reinforced soil (ODFS), randomly distributed fiber-reinforced soil (RDFS)

## Abstract

Incorporating sustainable materials into geotechnical applications increases day by day due to the consideration of impacts on healthy geo-environment and future generations. The environmental issues associated with conventional synthetic materials such as cement, plastic-composites, steel and ashes necessitate alternative approaches in geotechnical engineering. Recently, natural fiber materials in place of synthetic material have gained momentum as an emulating soil-reinforcement technique in sustainable geotechnics. However, the natural fibers are innately different from such synthetic material whereas behavior of fiber-reinforced soil is influenced not only by physical-mechanical properties but also by biochemical properties. In the present review, the applicability of natural plant fibers as oriented distributed fiber-reinforced soil (ODFS) and randomly distributed fiber-reinforced soil (RDFS) are extensively discussed and emphasized the inspiration of RDFS based on the emerging trend. Review also attempts to explore the importance of biochemical composition of natural-fibers on the performance in subsoil reinforced conditions. The treatment methods which enhances the behavior and lifetime of fibers, are also presented. While outlining the current potential of fiber reinforcement technology, some key research gaps have been highlighted at their importance. Finally, the review briefly documents the future direction of the fiber reinforcement technology by associating bio-mediated technological line.

## 1. Introduction

Soil is a porous media that exhibits weak behavior in tension, with geotechnical properties that vary with environmental factors. High demand has been made for unstable and erosive lands with poor geotechnical properties due to ever increasing population growth and urbanization. Therefore, development of effective stabilization techniques for these environmentally sensitive lands and soils has been called for [1,2].

In the past, there were myriad of soil improvement techniques have been proposed and implemented to stabilize the weaker lands prior to the constructions. The suggested improvement methods can be mainly categorized into two types: (i) mechanical methods of stabilization and (ii) chemical methods of stabilization. Mechanical methods include displacement and replacement, stage constructions, preloading, stone columns method, soil nailing and synthetic reinforcement applications. The chemical methods of stabilizing consist of deep in-situ mixing and surface stabilizations by using cement, fly ash, bottom ash, bentonite, gypsum, silica fume and blast furnace slag [3,4,5,6]. Also, the chemical stabilization techniques were widely incorporated with the ashes of several organic materials derived from burning process [7]. However, the above conventional stabilization techniques (mechanical and chemical) are coupled with severe environmental issues such as global warming via large carbon-dioxide emissions, high energy cost, environmental (air, land and water) pollutions, depletion of non-renewable resources and influx of heavy and dangerous substances to the geo-environment [8,9,10]. Therefore, ecofriendly applications are highly preferred in the field of Geotechnical and Geo-environmental engineering due to deliberation on healthy future of the globe. Thus, the current intentions of Engineers are targeting on modifying the existing weaker ground and soils using ground improvement techniques by ensuring sustainability in land use [10,11,12].

The concept and principle of reinforcement of soil using fibers was pioneered by Vidal in 1969, who found that adding reinforcing elements in a soil mass increases the shear resistance of the medium [13,14]. To date, nearly 4000 applications have been undertaken in more than 37 countries using the concept of soil reinforcement, after the invention by Vidal in 1969 [15]. Past research reflects an array of reinforcement materials ranging from low-modulus polymeric materials to high tensile strength metallic sheets, which were used as geosynthetics to enable the fiber-reinforcement of soil [2,16]. These conventional synthetic fibers were mostly the by-products of petroleum which is a non-renewable limited resource of the earth. Geosynthetic products have gained popularity due to their flexibility during processing, high specific stiffness and low cost [17]. Worldwide capacity of such plastic composites exhibited a massive increase from 0.36 million metric tons in 2007 to 2.33 million metric tons by 2013 and is expected to increase to 3.45 million metric tons by 2020 [2]. In addition, incorporating steel bars as soil reinforcement has been reported as a non-ecofriendly approach due to detrimental impacts to the environment at the end of its useful life as the corroded steel is very toxic to the environment [18]. Recently, “natural fiber-soil reinforcement” have gained momentum as one of the evolving sustainable soil strengthening techniques in geotechnical engineering due to its unique advantages such as environmental friendliness, resource abundance, minimal energy consumption, cost effectiveness and high potential over other established materials [12,19,20,21]. The rewards of this alternative are illustrated in Table 1 by comparing the energy content and cost between conventional synthetic materials and natural fibers [22]. The alternative of using natural fibers for conventional geosynthetic reinforcements to enhance sustainability has shown great potential and has attracted increasing attention in Geotechnical Engineering. Use of natural fibers remains a relatively new technique worthy of further study [12,15,21].

**Figure 1:**
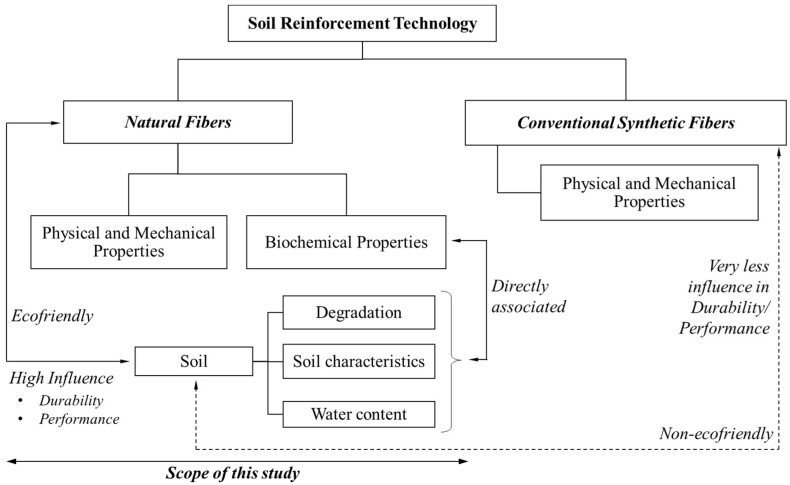
Schematic outline to understand the association between fiber properties and role of soil in fiber-soil reinforcement technology.

The prime objective of this state-of-the-art review is to explore the understanding of the emerging soil reinforcing technology coupled with natural fibers in the field of Sustainable Geotechnics. This review reveals the prime role of inherent properties of natural fibers on the performance and durability of fiber reinforcement at subsoil conditions as outlined in Figure 1. Also, fiber reinforcement technique and applications are distinguished based on the fiber reinforcing mechanism and clear characterization is presented. Further, the article presents the evolvement and existing level of soil reinforcement technology of different natural fibers with the aid of previous studies, hence methods to enhance the lifetime and reinforcement capabilities of fibers are presented. This review indicates some current research gaps in soil-natural fiber reinforcement technology and proposes potential directions of natural fiber reinforcement development and application.

## 2. Natural Fiber Materials

### 2.1. Characterization of Natural Fibers

Nowadays, natural fibers are widely incorporated in many engineering applications and industries including automotive industries, food and agricultural industries due to their abundance, sustainability, cost effectiveness, low density, favorable strength, stiffness and so forth. [2,23]. Primarily, natural fibers can be considered into three sections in view of their instigation: (i) plant fibers (bamboo, jute, coir, hemp, etc.), (ii) animal parts containing protein (silk, hair, wool, etc.) and (iii) minerals [23]. Based on the availability and applicability for large scale, geotechnical intentions have been projected towards plant fibers in terms of natural fibers [24].

**Figure 2:**
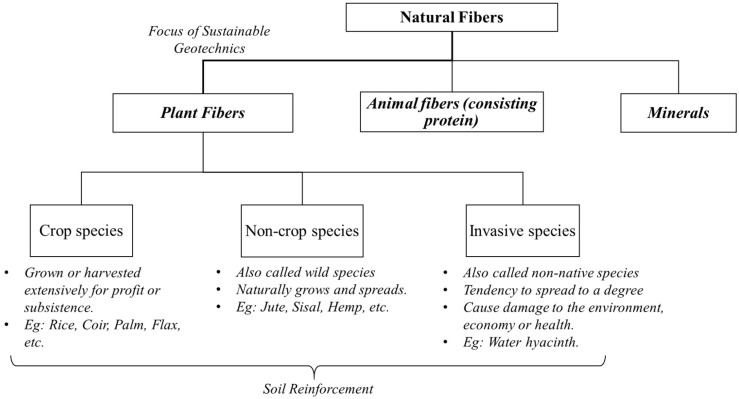
Schematic illustration for categorization of natural fiber incorporated in soil reinforcement.

Based on the economical aspect, plant fibers used for soil reinforcement can be classified into three categories: (i) crop species, (ii) non-crop species and (iii) invasive species, which are briefly exemplified in Figure 2. Such plant fibers may be originated from stem, leaf, seed, fruit, wood, cereal straw and other remains. However, what part of the plant the fiber originated from, the age of the plant and how the fiber is treated, are some of the aspects which influence the durability and performance of natural fibers. The wood fibers/debris of trees are not focused in this review as many practical limitations are associated while incorporating woody fibers for soil reinforcement. In fact, wood fibers have obtained a high momentum in composite industry of timber, concrete and other materials, hence scope of the woody fiber became lower in soil reinforcing intentions due to the following reasons: difficulties of frequent sourcing in large quantity, non-economic, low-flexibility of fibers and slow renewable compared to the species discussed in this review.

### 2.2. Biochemical Properties of Natural Fibers

**Figure 3:**
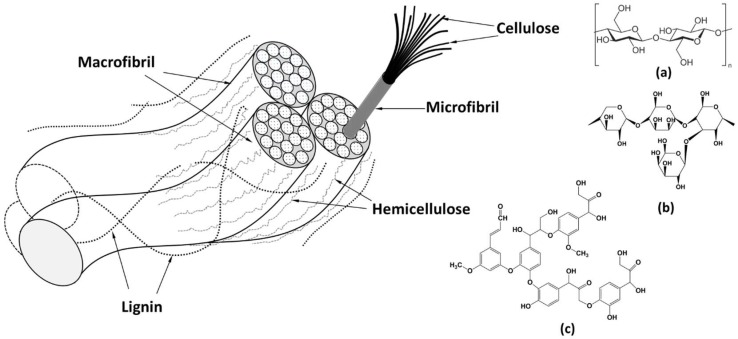
Fibril matrix structure of plant fiber and the chemical composition of (**a**) Cellulose; (**b**) Hemicellulose; and (**c**) Lignin.

Microstructurally, natural fibers can be defined as naturally occurring composites entailing mainly of hollow cellulose fibrils entrenched together by lignin and hemicellulose matrix [25]. Also, there can be the presence of pectins and waxes, whereas pectins provide flexibility for the fiber and the waxes make up the last part of the fibers and alcoholic compounds [24]. The cellulose fibrils (diameter about 10–30 nm) made of chained-cellulose molecules, are aligned along the length of the fiber, which afford higher mechanical (tensile and flexural) strength, in addition of providing rigidity [23]. Lignin plays important role as a protection layer which prevent the internal structure of fiber form degradation due to microorganisms. Cellulose is a biopolymer and content of cellulose and hemicellulose influences the moisture absorbent ability of the fiber structure [16]. The reinforcing efficiency and the behavior of natural fiber is linked with the nature of cellulose and its crystallinity as well [26]. A typical schematic diagram of fibril matrix structure (strands of cellulose molecules embedded in a matrix of hemicellulose and lignin) is given in Figure 3.

Some of the plant fibers which entails the potential of soil reinforcing, are summarized with their species, origins and biochemical compositions in Table 2. Higher cellulose content (>50%), which reflects the strength of the fiber, is observed in kenaf, hemp, flax, sisal, jute and bamboo fibers. However, the lignin which is an essential content for the fiber while reinforcing the soil in order to endure its durability, is comparatively poor (<10%) in flax and hemp whereas highest lignin content is found in coir fibers. Consequently, foremost biochemical compositions of both cellulose and lignin content clearly ensure the capacity of bamboo fiber in soil reinforcing compared to other natural plant fibers.

### 2.3. Physical and Mechanical Properties of Natural Fibers

The physical and mechanical properties are associated with the biochemical compositions of the fibers where cellulosic compound defines the strength of the fibers [2]. As designing phase of soil reinforcement has not been directly coupled with biochemical compositions, determination and interpretation of physical and mechanical properties of fiber are highly necessary in Geotechnical Engineering. The physical and mechanical properties of plant fibers related to soil reinforcement are summarized in Table 3. Kenaf, flax, bamboo and hemp exhibit higher mechanical strength parameters among the potential natural fibers, which is fundamentally due to their biochemical compositions. Moreover, the potential of plant fibers is illustrated in Table 4 by comparison to essential mechanical and physical properties of synthetic fibers.

## 3. Behavior of Fiber-Reinforced Soil

**Figure 4:**
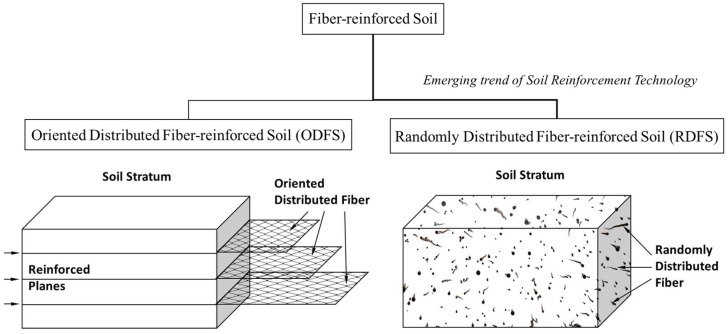
Classification and illustration of Fiber reinforcement mechanism of soil.

Soil reinforcement can be defined as a technique of improving the engineering characteristics and behavior of soil by introducing materials comprised of desired properties. The prime objective of reinforcing soil mass is to enhance its stability that is, shear capacity and bearing capacity, thereby to reduce deformations of soils [15]. Currently, the above reinforcing technique coupled with natural fibers has been rooted highly in Geotechnical Engineering. Global intentions have been magnetized towards natural fiber reinforcement technology due to its uniqueness, although limited effective life time of natural fibers in subsoil condition exists as a challenge [18]. Fibers imbedded in soil can be varied in forms, texture, stiffness, content, length or aspect ratio, orientation and so forth among which content, length and orientation of fibers are the most practical concerns in geotechnical applications [49]. Basically, fiber reinforced soil can be classified into two types based on their method of application: (i) Oriented Distributed Fiber-reinforced Soil (ODFS) and (ii) Randomly Distributed Fiber-reinforced Soil (RDFS) [2,20]. The schematic diagrams of ODFS and RDFS are clearly illustrated in Figure 4.

ODFS is the well-known reinforcement mechanism where natural fibers can be introduced by planner systems in vertical, horizontal or both directions. Generally, ODFS permits mechanical enhancements of natural fibers via modifications such as weaving, binding, combining or punching based on the requirements of applications. Mechanism of ODFS is similar to conventional geosynthetic approaches in which materials were introduced to weaker planes of the soil as geo-grids, geo-cells, geo-mats, geo-textiles and so forth. It is well understood that the ODFS technique mobilizes supplementary frictional strength along the fiber-reinforced planes whereas un-reinforced zones necessitate the survival by its own strength but possibilities still exist to generate failure planes through weaker un-reinforced zones [50].

**Figure 5:**
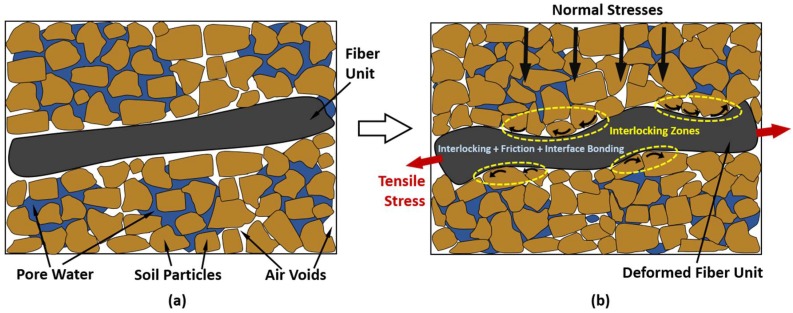
Schematic representation of a randomly distributed fiber unit at: (**a**) initial stage and (**b**) deformation stage due to loading, where the effect of interlocking, friction and interface bonding induces mobilization of the tensile stress on the fiber unit.

On the other hand, RDFS is a well-recognized soil improvement technique in which fibers comprised of desired property and quantity are assorted randomly and compacted in situ [2,16]. Incorporation of RDFS has become more popular in these days, as short discrete fibers can be simply added and mixed randomly with soil, much like cement, lime or other additives, thereby, it can provide isotropic increase in the strength of the soil composite without introducing continuous planes of weakness [51,52]. This RDFS method exploits the similar behavior of plant roots, which fortify the soil by contributing additional frictions and interlockings [53,54,55]. The mechanism of the RDFS is clearly illustrated in Figure 5. It is very clear that the distributed fibers subjected to tension contribute to the increase in strength of specimens [15,49]. Initially, soil particles subjected to stresses attempt to densify, which persuades deformation of fiber material, subsequently direct forces are generated on fiber at fiber-soil interlocking stage due to rotation and direct impact of soil particles (Figure 5). Simultaneously, soil particles which are in contact with fiber, induce to develop frictional forces on fibers in addition to interlocking forces [2]. Eventually, the interlocking forces coupled with frictional forces tend to mobilize the tensile stress on fiber material. Moreover, random distribution of fibers mobilizes fiber-soil adhesive bonding, which utilizes additional composite strength and the interaction of the flexible fibers behaves as a structural mesh that holds the soil together increasing the soil structural integrity [15,49].

Fiber length is one of the variable factors impacting the degree of fiber reinforcement in improving soil behavior. The influence and mechanism of fiber length are basically measured by the concept of aspect ratio (η), defined as the ratio of fiber length (l) over fiber diameter (d), expressed in Equation (1),

(1) $$\eta \;=\;\frac{l}{d}$$

Generally, fiber length acquires more significant role than fiber diameter in RDFS engineering practices. As naturally diameter of fiber exists inherent with a very low significance in standard deviations, enhanced strength of the reinforced soil is vitally captured by length of discrete fibers which directly influences the interactions in soils [49].

## 4. Soil-Natural Fiber Reinforcing Applications

Globally, several researchers have tried to exploit the strengthening characteristics of natural plant fibers. However, a consensus of geotechnical approaches is lacking due to variations in local production, consumption and incorporation of fiber materials. This review attempts to expand our understanding of soil reinforcement potential of each plant fiber by relating the application modes of fiber materials to soil characteristics and their interactions.

### 4.1. Bamboo Fiber

Bamboo is an abundant and viable natural resource and there are more than 1250 species worldwide ranging from small diameter “reed like” bamboo to large diameter “woody” bamboo [24,56]. Growth rate of bamboo ranges from 30 to 100 cm per day in growing season and requires only 3–5 years to be mature enough for harvesting [28,57,58]. Due to high water-absorption properties, high flexibility, high intensity, high fiber content, low weight, low costs, fast-growing rate, perennial and asexual culms, bamboo has gained the global potential as a civil engineering material in broad field of applications [59,60]. In the applications of Architectural Engineering and Composite Material Engineering, bamboo has been involved as reinforcing element to replace the conventional materials including steel and polymer plastics due to cost effectiveness and sustainability consensus [8,40,61,62,63]. In the field of Geotechnical Engineering, bamboo has been utilized in various scopes and applications such as soil reinforcement, slopes protections, prevention of soil erosion, bearing capacity enhancement and many other ecofriendly approaches [64]. Studies that attempted to exploit the applicability of bamboo fiber for soil stabilization are critically reviewed in this section by distinguishing the distribution mechanism of fibers under ODFS (bamboo as grids, cells, mats/mattress and geobamtile) and RDFS (bamboo as powder, chips, flakes and roots).

#### 4.1.1. Bamboo ODFS Applications

**Figure 6:**
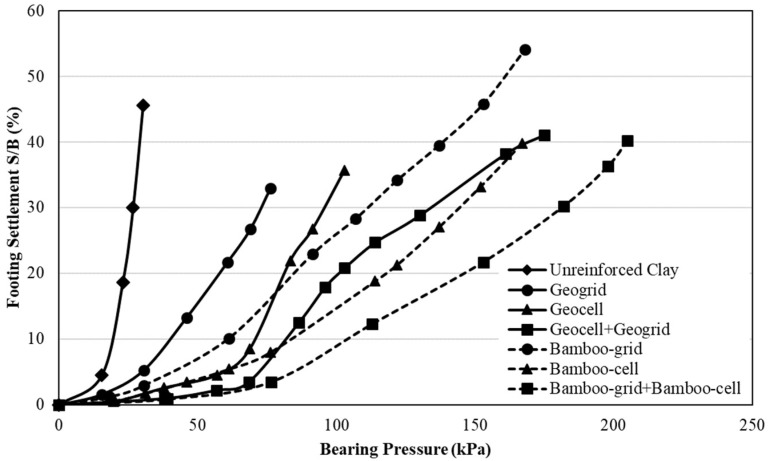
Comparison of bearing pressure-settlement curves of different bamboo oriented distributed fiber-reinforced soil (ODFS) reinforcement techniques (data sourced from reference [65]).

Grids consist of two-dimensional planer nature where fibers may be oriented either one way or two ways of that corresponding plane, whereas cells consist of three-dimensional nature, offers all-round confinement to the encapsulated soil. Generally, tensile strength of bamboo-grids are about nine times higher than that of commercially available geo-grids [65,66]. Also, bamboo-grid exposes surface roughness up to 3.5 times higher than that of conventional polymer geo-grids hence leading to higher pull-out resistance [67,68]. Bamboo-grid constructed with 1 cm diameter bamboo rods, would be adequate to control the settlement of sandy silt by 20–30% whereas settlement control by conventional geo-grid (made of high tenacity polyester) is only 10–15% [69]. At the same time, bamboo-grid controls the settlement and lateral deformation of soft clay by around 21% and 31% respectively [70]. Normally, bearing capacity of soil increases with number of grid layers applied within failure envelope and bamboo grid (one way oriented) exhibits the lowest settlement and highest bearing capacity when the reinforcement layer is placed at the depth of 0.30 B below the foundation, where B is foundation width [71]. Moreover, Hegde and Sitharam [65] have undertaken an investigation to compare the reinforcing effect of grids and cells (250 × 210 mm) placed at 0.3 B and 0.1 B (optimum depths) below the footing (B = 150 mm) in soft clay. The comparison of bearing capacity behavior of grids and cells are presented in Figure 6.

It can be observed that the bearing capacity of the clay bed reinforced with bamboo-grid shows 1.3 times more than that of clay bed reinforced with geo-grid. Generally, incorporating bamboo-grid or bamboo-cell can increase the ultimate load bearing capacity of soft soils up to six times higher than that of unreinforced soil [65,66,69,72]. However, combination of bamboo-grid and bamboo-cell is recommendable than incorporating them alone in subsoil engineering.

Sai and Heng [72] have extended the bamboo-grid concept as Geobamtile, is a three-dimensional bamboo grid-frame buoyant system, which has recently been commercialized and adopted by the construction industry of Malaysia so as to control excessive settlement of soft soil ground including organic and peaty lands, where the matured bamboo culms are formed in a large scale of grids as an extensive raft system and a fabricated grid would be placed in a critical weaker subgrade. Toh et al. [73] have introduced a bamboo-fascine mattress by combining nonwoven geotextile sheet with bamboo grid to enable a stable area (30,000 m^2^) for human access over a fibrous peaty land by controlling mud waves. Bamboo network technology combining geobamtile and bamboo-mattress (woven) enhances the bearing capacity of hydraulic fill soft soil by 695% after three months from the process [74]. Incorporating bamboo-mats not only controls the differential settlement effect but also limits the settlement of marine beach soil with 15 mm [75]. Although, bamboo ODFS technique has successfully rooted in geotechnical applications, many investigations in soft soil have resulted only a limited bearing capacity improvement, which is not adequate for the Engineering constructions [66,76].

#### 4.1.2. Bamboo RDFS Applications

In the recent past, bamboo has gained the tendency of RDFS application in Geotechnical Engineering field, whereas fibers are randomly mixed with soil for improvement purposes. Generally, bamboo powder, chips and flakes are the most common fiber modes used in RDFS applications and researches have concluded that RDFS technique increases the rigidity modulus, unconfined compressive strength (UCS), shear properties and California Bearing Ratio (CBR) value, besides decreases the dry density and settlement of the soil [51,77]. Basically, water absorption of bamboo fibers increases with reducing size due to surface area effect [78]. But, water absorption of bamboo flakes is significantly higher than bamboo chips and the initial absorption of flakes is around 6 times higher than the chip [64]. However, the absorption of both chips and flakes reduces with the time [64,78]. Further, investigations on the effect of fiber amount and fiber size on the shear strength improvement of sand have been performed by Devi and Jempen [79]. It has been detailed that the shear strength parameters (c′ and φ′) of the fiber reinforced soil obtained their optimum when the amount of fiber addition increases to 4% and the shear strength increment has been noticed when the length of fibers increases from 20 mm to 30 mm at a stable diameter ranging between 1 and 2 mm. Shigematsu et al. [80] have extensively investigated the applicability of bamboo chips RDFS as pedestrian pavement material (Sand, D_50_ = 0.65 mm). It has been concluded that UCS, stiffness and permeability of reinforced pavement increase favorably with increasing bamboo content. Although, CBR decreases with increasing bamboo content, 10% CBR is ensured even at 80% of bamboo content. There are also instances of reinforcing in order to enable the stability of slope [81,82]. Bamboo roots RDFS contributes to increase shear strength and exhibits up to 55% additional peak shear strength with 5% soil-root volume ratio, whereas diameter of roots ranged from 3 to 10 mm [81]. Moreover, RDFS composed of bamboo chips shows a significant improvement in mitigating erodibility potential of cut slopes [82].

At the same time, attentions of researchers are preferably focused on improving the behavior of challenging soft soils by randomly distribution of bamboo fibers. Sato et al. [83] have extensively investigated the improvement of soft clay by incorporating bamboo chips and flakes at the consideration of moisture absorption characteristic of bamboo material. However, studies conclude that improvement of soft soils by incorporating bamboo fibers alone (RDFS) is limited to only a certain level due to the absence of bonding/solidifying material [78,83]. Thus, approaches extend the stabilization of soft soil by adding a small amount of cement material to bamboo RDFS in order to enhance the reinforcing effect significantly [64,78,83,84].

### 4.2. Jute Fiber

Jute is one of the most common natural fiber crop being cultivated in the world, which is relatively inexpensive and commercially available in the required form [12,29]. It is widely available in Asian Countries as well as Brazil and generally grows to 2.5–4.5 m height [37]. Jute fibers have been found to be effective in improving geotechnical characteristics of soil and are being used extensively in many applications such as consolidation of soft soils, rural road construction, protection of river banks, stabilization of embankments, erosion control, management of slopes and so forth [21].

Although more attentions have been paid on jute RDFS, few researchers focused on applicability of jute ODFS incorporating jute-textiles. Application of woven jute-textile has been encountered as an economic and reliable scheme in drainage applications of preloading as well as consolidation applications in soft soil formations [85,86]. Particularly, open weave jute-textile is widely known as erosion-controller due to its triple enhanced properties: controlling the flow velocity, moisture absorption and lucidity to vegetation [21,87]. Intrinsic assembly of jute-textile consists about 40% direct cover of jute yarns and 60% of open area, which helps to control the velocity of ground water flow thereby severely inhibiting the detachment and transport of soil [87]. Jute-geotextile also comprises major role as soil saver: interface separator to prevent soil or sand loss in geotechnical applications and minimal soil loss via jute-sand composite layer has been observed for an upper bound water content of 200% [85]. Moreover, glued/punched jute-fabric has been known as a better jute-textile material which exhibits higher tensile strength (about 30%) compared to that of jute reef consisting ten yarns [12]. However, treated jute-geotextile would lose its 50% of tensile strength after 1080 days due to UV, moisture related weathering and biodegradation in a tropical field installation environment and which is about 3–5 times longer than those reported for untreated jute geotextiles [88].

On the other hand, strength behavior of jute RDFS in clayey soil has been examined with different dosages (0.2–1%) and different lengths of RDF (5–20 mm) and optimum strength value (CBR) which is about 2.5 times higher than that of native clay has been observed at the dosage and length of 0.8% and 10 mm respectively [9]. At the same time, jute RDFS can be able to raise the CBR value by about 50% compared to that of unreinforced residual soils [89,90]. In addition, jute RDF plays a beneficial role in reducing the post peak strength loss of expansive soil by enhancing strength parameters and stress-strain behavior. Effective content and length of jute fibers which provide the optimum shear strength parameters in expansive cohesive soils have been concluded as 0.6% and 12 mm respectively, whereas shear strength (τ) of reinforced soil have been correlated with normal stress (σ) as given in Equation (2) [49].

(2) τ=0.478 σ+126.5

Moreover, it is broadly recognized that fiber orientation influences reinforcing effects significantly [91] and few researchers have investigated the orientation effect of jute fibers by triaxial laboratory experiments on granular soils with different fiber orientation angles. Michalowski and Cermak [92] have introduced a simple equation—Equation (3)—to evaluate effective orientation angle ($\theta_{0}$) in cohesionless granular soils based on the numerical analysis. Recently, Diambra et al. [93] have proposed Equation (4) to obtain effective orientation angle, based on zero strain condition and Mohr’s circle in terms of major and minor principal stresses, hence simplification of Equation (4) leads to Equation (5).

(3) $$\theta_{0}=\;\pm \arctan\sqrt{\frac{K_{p}}{2}};\;\;K_{p}=\;\tan^{2}(\frac{\pi }{4}+\;\frac{\varphi }{2})$$

(4) $${\dot{\varepsilon }}_{1}\sin^{2}\theta_{0}\;+\;{\dot{\varepsilon }}_{3}\cos^{2}\theta_{0}=\;\dot{\varepsilon }\;\theta_{0}=0$$

(5) $$\theta_{0}=\arctan\sqrt{-\;\frac{{\dot{\varepsilon }}_{3}}{{\dot{\varepsilon }}_{1}}}$$

where, $\theta_{0}$ is inclination of zero incremental strain direction; ${\dot{\varepsilon }}_{1}$ and ${\dot{\varepsilon }}_{3}$ are major and minor principal strain rates of specimens respectively; φ is internal friction angle. The fibers aligned within a range of $-\theta_{0}\;\leq \;\theta \leq +\theta_{0}$, lead to be effective in improving soil strength, whereas fibers aligned out of the range result in an adverse effect in cohesionless soils. On the other hand, horizontal fibers are most favorable in cohesive soils, as peak shear stress decreases with increasing orientation angle from 0 to 90° [49]. There are also instances of stabilizing fine-grained soil using jute fibers by introducing lime as cementation agent and the optimum effect of jute and lime (0.75% and 4%) causes about 15% of strength (UCS) increment [94,95].

### 4.3. Coir Fiber

Coir fiber is the material between hard-internal shell and outer coat of a coconut, generally diameter ranges 0.1–0.3 mm and length ranges 10–50 mm [96]. It comprises a large amount of hydroxyl group, which makes coir fiber hydrophilic in nature hence leads for higher moisture absorption and weak interfacial bonding while using as reinforcement [97]. Although coir fibers are reported to have good tensile strength and stiffness, exposure to chemical environment, hydrolysis, oxidation or dehydration results significant strength reduction [31,97], thus limited researchers have focused towards the application of coir fibers in the field of Geotechnical Engineering up to now. Among them, only a few researches have been focused on applicability of ODFS as coir-textile manufactured from coir fibers. Coir-textile has been implemented as a shielding material for slope soil as to protect the soil from erosion [31,98]. However, quicker biodegradation of coir fiber (22% of tensile strength only remains after 7 months from application), tends to drop the tendency of coir-textile applications.

On the other hand, coir RDFS has attracted the intentions on soil reinforcing applications these days. Basically, percentage of water absorption and tensile strength of coir reinforced soil increase with amount of coir content. However, treating the coir fibers with both NaOH and CCl_4_ prior to the application enhances the reinforcing effect in clayey soil, which raises the strength significantly with increasing coir content up to 1.6% [99]. Also, studies widely acknowledged that the overall behavior of reinforced soil depends not only on the optimum quantity of coir fiber but also quality of treated coir fiber [14,97]. Coir RDFS increases the strength (CBR value) of expansive soft soil favorably by 335% at optimum 0.6% short coir fiber content [100]. Also, the optimum shear as well as UCS behavior of silty sand has been observed at the coir fiber content of 0.75% [51]. Further, coir fiber reinforcement increases the CBR value of granular subgrade by 96% [89]. However, CBR value increases up to coir fiber content of 10% and further increment of fiber quantity declines the strength of reinforced soil [101]. At the same time, reinforced soft soil combining coir fiber with small addition of lime exposes higher tensile and UCS at the 1% of optimum coir fiber content [102]. Similar optimum content (1%) of coir has been resulted, while executing coir fiber together with small amount of cement and pond ash [97]. Moreover, lateritic soil stabilization of low volume pavements suggests that addition of 1% coir fiber along with 3% of cement by weight of soil significantly increases the UCS and CBR values [14].

### 4.4. Palm Fiber

Oil palm belongs to the species Elaeis guineensis, which originated from tropical forests of West Africa [52]. So far, only very few studies have focused on the applicability of palm fibers in soil reinforcement techniques. Dry density and optimum moisture content of palm RDFS, decrease with increasing fiber content due to a low unit weight of palm fibers [18]. Palm random fibers reinforced soft clay exhibits optimum shear behavior at fiber content of 0.75% [103]. At the same time, silty sand reinforced with palm fibers of 30 mm reveals optimum shear behavior at fiber content 0.5%, which increases the friction angle and cohesion about 25% and 35% respectively [52]. However, optimum CBR value of silty sand has been reported at fiber content of 0.75% of 40 mm fiber length and obtained penetration strength is 2.6 times higher than that of unreinforced soil. Moreover, the optimum palm fiber content of non-cohesive sandy soil has been reported as 1.00% and obtained CBR of reinforced soil is around six times higher compared to unreinforced sandy soil [18].

### 4.5. Sugar Cane Bagasse Fiber

Bagasse fiber is a waste material of the sugar cane industry, leftover after extracting the sugar cane juice and existing as rich resource due to its high yield and annual regeneration capacity [47,104]. Sugar cane bagasse fiber has been just introduced in Geotechnical Engineering applications and only a very few studies on bagasse fibers reinforced problematic soil have been performed so far. Bagasse has been identified as one of the potential material to stabilize the expansive soils [46,105]. Bagasse fiber reinforced clayey soil exhibits a better control in shrink-swell behavior at the 2% optimum content of fibers (diameter ranges 0.3–3.1 mm, length ranges 0.3–13.8 mm). At the same time, combination of bagasse and lime (optimum of 1.5% and 6.25% respectively) extends the UCS by around 145% and reduces linear shrinkage effect compared to the reinforcement by bagasse fibers alone [46].

### 4.6. Water Hyacinth Fiber

Water hyacinth is one of the world’s most invasive species and it has amassed global awareness due to its sooner spread and mobbed growth [16]. The management of this weed is reported to be costly and value addition of this waste weed would be a beneficial endeavor. As an alternate way of waste management, few geotechnical studies have been raised on water hyacinth fiber. Application of Water hyacinth woven geotextile (ODFS) up to two layers in silty sand has been investigated and the observations suggest that reinforced soil exhibits CBR penetration strength two times higher than that of unreinforced soils. Though penetration strength increases with number of geotextile layers, increment rate reduces [26]. Investigation on soil erosion control by using water hyacinth has concluded that runoff was reduced when using water hyacinth cover combined with grass, which was further reduced with increasing growing periods of grass [106].

### 4.7. Rice Husk Fiber

Rice husk is an abundant food waste with low price, biodegradability potential [107]. The composition of rice husk is more complex than other fibrous material, silica is 91.1% distributed in rice husk and occurs as hydrated grains, which are biosynthesized through the polymerization of silica acid by living organisms [107,108]. To present, there is only a limited study focused on applicability of rice husk fiber for soil reinforcing. Studies suggest that rice husk powder additives and curing duration influence a significant effect on the strength of reinforced soil. Rice husk fiber powder content of 15% with regard to 3-days curing, has been stated as optimum combination to obtain UCS of rice husk reinforced fine grained soil [109].

### 4.8. Sisal Fiber

Recently, sisal plant has been recognized as a potential engineering material due to its strength, durability, ability to stretch, resistance to deterioration [110]. Up to now, only a limited number of researchers have focused on the applicability of sisal fibers in Geotechnical applications. Generally, addition of sisal fibers increases the ductility of soil without significant effect in compressive strength [111]. The optimum length and content of sisal fibers in RDFS method have been concluded as 20 mm and 0.75% respectively, cohesion of soil has been raised by 265%, whereas linear variation is observed between cohesion and fiber content. However, increase of fiber length and content linearly decreases the maximum dry density (MDD) and optimum moisture content (OMC) [112]. Also, inclusion of sisal random fibers exhibits better improvement of shear and deformation characteristics in silty clay [113].

### 4.9. Other Fibers

Banana fiber is found to be a good reinforcement in polyester resin applications, which attracts the intentions for the potential of soil reinforcement applications [110]. Also, hemp can grow to 4 m in just 12 weeks, fiber ranges in length from 1.0–2.5 m, which ensures the potential for reinforcement applications [36]. Although the potential of many fiber species (flax, banana, hemp, kenaf) has been identified based on the biochemical and mechanical properties, soil reinforcing applications of such fibers have not been performed yet.

Straw fibers (barley, wheat, pine) are claimed to be the most cost-effective soil reinforcing material. However, relatively few published data are available on its performance as reinforcement to soil. The shear strength of reinforced clayey silt has been optimized at 1% of 10–500 mm length barley straw RDFS [15]. Pine straws plays a major role as a perfect ground cover for soil stabilization and erosion control projects, as interlocking benefits of pine straws allows the straw not to float away and holds soil in areas where grass cannot grow [20,48]. Although many forage grass fibers are being focused and harvested as straw in agriculture and food industries, they have not been investigated as soil reinforcing medias up to now.

## 5. Effect of Water in Fiber-Reinforced Soil Behavior

Soil matrix is a three-phase (soil particles, water and air) media hence the stresses and deformations are taken by all three phases. Shear strength of a soil can be defined as the maximum shear resistance that can be mobilized within a soil mass without any failures. In nature, soil matrix can exist in two conditions: (i) fully saturated condition and (ii) unsaturated condition. Terzaghi [114] proposed the Mohr Coulomb equation given in Equation (6), to provide the shear strength in terms of effective stresses for the saturated soil, in which all the pores filled with water only. But, the natural surficial soil deposits of large area of the earth are unsaturated soils comprised of water and air voids due to the influence of climate and vegetation. The presence of continuous air phase and rendered pore water phase tends to differ the principles and concepts involved in saturated soil theory. Thus, Fredlund and Morgenstern [115] extended the constitutive equation given in Equation (7) for unsaturated soils providing two independent stress state variables including net normal stress and matric suction.

(6) $$\tau =c^{\prime }+\;(\sigma -u_{w})\tan(\varphi^{\prime })$$

(7) $$\tau =c^{\prime }+\;(u_{a}-u_{w})\tan(\varphi^{b})+\;(\sigma -u_{a})\tan(\varphi^{\prime })$$

where, τ = shear strength; $c^{\prime }$ = effective cohesion; $u_{w}$ = pore water pressure; $\left(\sigma -u_{w}\right)$ = effective stress; $\varphi^{\prime }$ = effective friction angle; $\left(\sigma -u_{a}\right)$ = net normal stress; $\left(u_{a}-u_{w}\right)$ = matric suction; $\varphi^{b}$ = angle of frictional resistance due to matric suction; $u_{a}$ = pore air pressure.

As illustrated in previous sections, shear strength of fiber reinforced soil is primarily generated from interactions of soil-soil and soil-fiber. Now, it is well-understood from Equations (6) and (7) that the shear strength of reinforced soil is mechanically governed by the water content presenting in subsoil conditions. In addition, presence of moisture on the particle interfaces roles as a lubricant layer, which tends to induce slipping and rupture effect, hence reduces the friction and cohesion in reinforced soil [49]. Also, it has been concluded that slipping is the prime failure mechanism of fiber reinforced soil, which induces more at the presence of water content [116].

**Figure 7:**
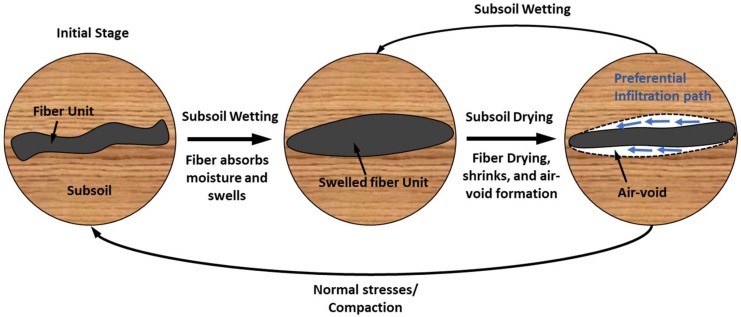
Schematic illustration of the shrink-swell cycles of fiber at subsoil condition and formation of infiltration path along interface voids.

Moreover, natural fibers comprised of holocellulose (cellulose and hemicellulose) exhibit hydrophilic behavior, which plays a vital role in fiber subsoil behavior. In subsoil conditions, fibers are liable to undergo cycles of wetting and drying and tends to expose shrink-swell behavior [2,15]. As shown in Figure 7, fiber absorbs water due to the internal osmotic pressure and expands during the subsoil wetting. Same fiber tends to shrink at subsoil drying era, which leads to affect the interaction of fiber-soil interface due to the formation of voids around it (Figure 7). Instantly, matric suction develops in the generated air void zone, which tends to enhance the shear strength of reinforced soil based on the relationship presented in Equation (7). However, inadequate compaction of reinforced soil and large quantity of fibers coupled with the air void formations stimulate the preferential infiltration path along such interface voids of reinforced soils [2], which creates an adverse effect on the stability of reinforced soil.

**Figure 8:**
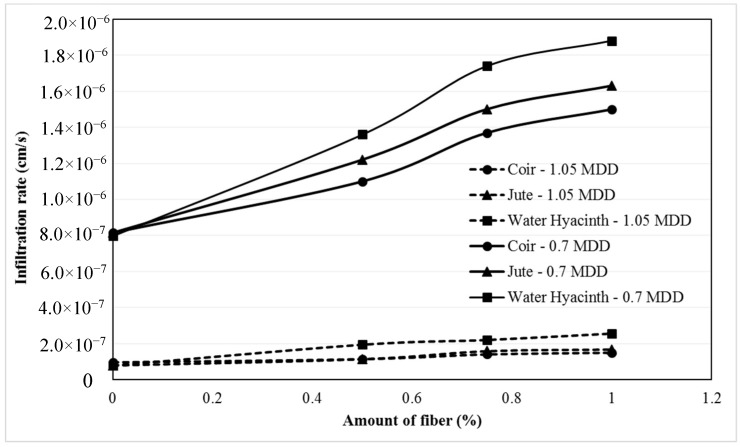
Effect of compaction on infiltration characteristic of fiber reinforced soil: comparison between different fiber-soil composites (data sourced from reference [16]).

Investigations have been performed by Bordoloi et al. [16] to study the above infiltration effect using different fibers (coir, jute and water hyacinth) at different maximum dry densities (MDD). The comparison based on the results are presented in Figure 8, hence it is ensured that all the fibers enable add-on void paths with increasing fiber content, thereby increase the infiltration rate. However, MDD highly governs the infiltration effect, where effect is significantly reduced at higher MDD of 1.05 in all fiber cases. Thus, greater compaction work is highly recommended to obtain high dry density, which mitigates the effects of voids and water lubricant layer, so that to enhance fiber reinforcing engineering practices.

## 6. Fiber Degradation and Recommended Treatments

Although fibers comprise many desirable engineering properties, degradation due to microorganisms remains as a major challenge in fiber soil reinforcement technique [41,117]. Such biodegradation of fibers results depletion of strength due to breakdown of cell wall polymers (cellulose, hemicellulose and lignin). Basically, moisture absorption effect and biodegradation are interconnected, as moisture absorption resulted by holocellulosic content provides favorable conditions for microorganisms to live on the fiber material [2]. However, composition of lignin which exists as the outer layer of fiber matrix, resists the intrusion of bacterium hence protecting the fibers up to a certain extent [118]. Moreover, the biodegradation happens in both aerobic and anaerobic conditions, whereas lignocellulose breaks down into residue carbon polymer and reactions of aerobic and anaerobic conditions are presented in Equations (8) and (9) respectively [2].

(8) $$C_{\mathrm{polymer}}+{\;O}_{2}\;\overset{\mathrm{aerobic}}{\to }{\;C}_{\mathrm{residue}}+{\;C}_{\mathrm{biomass}}+{\;\mathrm{CO}}_{2}↑+{\;H}_{2}O$$

(9) $$C_{\mathrm{polymer}}\;\overset{\mathrm{anaerobic}}{\to }{\;C}_{\mathrm{residue}}+{\;C}_{\mathrm{biomass}}+{\;\mathrm{CO}}_{2}↑+{\;\mathrm{CH}}_{4}↑+{\;H}_{2}O$$

The degradation which heavily affects the durability of natural fiber in subsoil environment, which depletes the reinforcing effect of fiber, is often regarded as a challenge need be avoided. As to ensure the long-term durability of fibers, many treatments have been undertaken to alter unfavorable characteristics of fibers and are briefly summarized in Table 5.

Basically, alkaline treatment disrupts the lignin structure of the fibril matrix and roughens the surface of the fibers by replacing the hydrogen bonding as given in Equation (10) [124].

(10) $$\mathrm{Fiber}-\mathrm{OH}\;\overset{\mathrm{Alkaline}/\mathrm{NaOH}}{\to }\;\mathrm{Fiber}-O-\mathrm{Na}+{\;H}_{2}O$$

(11) $$\mathrm{Fiber}-\mathrm{OH}\overset{{\mathrm{CH}}_{3}-C\left(=O\right)-O-C\left(=O\right)-{\mathrm{CH}}_{3}}{\to }\;\mathrm{Fiber}-{\mathrm{OCOCH}}_{3}+{\;\mathrm{CH}}_{3}\mathrm{COOH}$$

Acetylation treatment process enhances the plasticization of lignocellulosic fibers by substituting polymer hydroxyl groups by acetyl groups as shown in Equation (11) [125]. Additionally, the fiber cell walls which have achieved the hydrophobic characteristic tends to exhibit more adhesive behavior within soil matrix [126]. Also, permanganate treatment process reduces the hydrophilic tendency of fiber due to the graft copolymerization of cellulose matrix induced by activated Mn^3+^ ions. Although many methods of chemical coatings have been proposed to achieve an extra layer of protection to the fibers, direct chemical modifications illustrated above are being used extensively in RDFS applications [2]. However, due to the high lignin content, bamboo fiber owns a uniqueness, which can be applicable to soils without any prior treatments as it is seldom affected by pathogens during its design life of 10–15 years [15,56,60].

## 7. Future Prospects

These days, geotechnical profession has been motivated by the sustainability tendency of natural fiber soil reinforcement technique, which is particularly essential as the profession lies at the edge of the natural and built environments and can significantly impact the economy, society and environment. Based on the extensive discussions by focusing inherent properties and applications of each potential natural fiber with the aid of previous studies, some key research gaps have been identified, hence the future prospects of research in the area of natural fiber soil reinforcement are listed below.

It is well understood that RDFS has successfully executed its potential in Geotechnical Engineering, whereas fortifying of soil is primarily influenced by the inherent biochemical properties of natural fibers. Although many plants have reached the application stage as fibers in RDFS, some potential fibers (flax, banana, hemp, kenaf, barley, wheat), which are laid out in this review, have not been performed in RDFS up to this point. It is beneficial, if some researches attempt to focus the above fiber materials in future.

As discussed in previous sections, biochemical properties of fibers play the vital role in functioning of lignocellulose fiber as soil reinforcing material. However, only a very few studies have been focused to investigate the biochemical properties of fiber materials in geotechnical engineering and further attentions have to be paid on this query.

Natural fibers are hydrophilic and biodegradable, which create limitations in geotechnical engineering practices [49]. To date, biochemical compositions of fibers are being used as relative indicators of degradation and hydrophilic tendency. However, there is no clear understanding or relationships between effective lifetime of fiber material and biochemical compositions yet. Therefore, the review recommends correlating the quantity of lignocellulose (lignin and hollocellulosic) compounds with the durability/life period of fibers, which would be highly beneficial for fiber soil reinforcement designs as well as for field applications in the Civil Engineering field.

One of the very important factor in durability which has not been investigated in detail up to now is the fatigue response of fiber materials. Review prioritizes to evaluate the fatigue responses of natural fiber materials at different possible subsoil conditions including cyclic stress-strain, wetting-drying and freezing-thawing responses as future work.

It is very clear that surface roughness plays a significant role in sourcing the fiber-soil friction. Quantifying the surface roughness of natural fiber materials is highly necessary to relate the effect of surface roughness with stability of reinforced strata. Since there are no comprehensive studies investigated on this context [2], review suggests not only to perform the pullout-frictional investigations but also to extend the experimental studies for failure mechanism of reinforced fibers.

The review attempted to illustrate the mechanisms of chemical treatments and listed the treatment modes adopted to enhance the durability and performance of fiber materials. However, understanding regarding enhancement in effective lifetime of fiber material after treatment process is still remains as an unknown, thereby it is recommended for the future studies to consider the effective lifetime of treated fibers comparing with that of untreated, which would be really beneficial in selecting appropriate treatment process based on applications.

The review provides evidence that the number of chemical treatments investigated and developed to date are limited, thereby this review recommends promoting and advancing treatment methods to improve the repellency and durability of fiber materials to different subsoil challenges.

Also, review clearly reveals that there is a considerable research gap due to the absence of large-scale investigations on fiber-soil reinforcing technique, as most of the investigations performed up to now are small-scale laboratory studies. But, it is very essential to undertake large-scale investigations at the evolving stage of the reinforcement technique to experience the scale and boundary effects [15]. Therefore, review highly recommends to scale-up the future investigations in order to promote the reinforcing mechanism.

Further, it can be stated that the cost of the naturally derived fibers is low compared to the man-made synthetic fibers. However, more studies are required to analyze the cost for the product developments, field applications, monitoring and performance evaluation in order to quantify the economic benefits of this green approach. Moreover, RDFS technique reveals the potential on soft soil improvement, as fibers afford the adequate initial strength to the soil, subsequently soft soil gains the strength due to the process of consolidation by the time fiber breaks down and the reinforcement effect may not be required for longer term [68,127]. However, soft soil improvements are bounded within a specific range, which is due to the absence of bonding/solidifying agent in fiber reinforced soil [15,78]. Thus, review recommends a possible hybrid study combining RDFS technique with “bio-mediated soil improvement technique” known as “bio-cementation” which has already emerged in soil stabilizations, where microbial precipitated calcite tends to act as the bonding agent between soil particles [128,129,130,131,132]. Among the emerging trends of geo-technologies, a new potential and essential pathway is directed for the future scope, which would be highly beneficial for developments of the future.

## 8. Concluding Remarks

Sustainable consensus in Geotechnical Engineering intends to replace the conventional synthetic materials by natural plant fibers nowadays. The current review attempts to bring out the understanding of soil reinforcing technique using natural plant fibers. Based on the critical review, the following conclusions have been drawn.

Behaviors of plant fibers are inherently different from the conventional synthetic materials, whereas biochemical properties govern the functioning of fiber as reinforcing material. Based on the compositions of both cellulose and lignin content, bamboo fiber comprises the foremost soil reinforcing capacity and durability compared to the other potential fibers focused in this review. Based on the application mechanism, fiber reinforcing practices can be considered as ODFS or RDFS. However, desirable benefits of RDFS have enthused the current trend, thereby many natural fibers have gained the tendency for RDFS applications presently. Bamboo RDFS of 20–30 mm shows optimum strength behavior of granular soil at 4% of fiber content in wide range of applications. At the same time, jute RDFS of 10 mm shows optimum behavior of soft soil at 0.8% of fiber content. Coir fibers are relatively weak in durability due to quick degradation and 0.6% of fiber content exhibits the optimum behavior of soft soils. Sugarcane bagasse RDFS consist of high potential in expansive soil stabilizations, which exhibits optimum performance at 2% of fiber content. In addition, palm and sisal RDFS consist adequate capacity in soft soil stabilization at 0.75% of optimum fiber content. Accordingly, RDFS technique consists of great potential in soft soil stabilizations and remains to be promoted further in future.

Understanding of behavior of fibers at various subsoil conditions is highly essential for reliable stabilizations. Hydrophilic and biodegradable characteristics of fibers coupled with frequent presence of moisture creates a challenging environment, which affects not only the fiber-soil interactions but also durability of fibers. Shrink-swell behavior of fibers at subsoil conditions stimulates the infiltration paths along the interface voids, hence induces the slipping and failures at contact zones. Performing an adequate compaction work of 1.05 MDD, is one of the effective way to control the issue due to water lubricant layer in soil reinforcing applications. In addition, modifying adverse characteristics of fiber materials by chemical treatments can promote the fiber-soil behavior as well as lifetime of fibers. Alkali treatment, acetylation treatment and permanganate treatment are the widely used methods to enhance the performance by modifying molecular structure of fiber materials. At the same time, bitumen, ABS thermoplastics, nano-clay and resins are being incorporated to shield the fiber materials externally. However, selection of treatment method should be executed based on the fiber material and application target.

Based on the review, key research gaps have been pointed out and useful suggestions and recommendations have been given for the future development and promotion of natural fiber-soil reinforcement technology. In sustainable engineering point of view, this soil stabilization technique is not only an effective resource/waste management approach but also helps to create myriad of job opportunities globally.

## Figures and Tables

**Table 1:** Comparison of specific properties and cost of conventional synthetic fiber and natural plant fiber materials.

Fiber Material		Cost (USD/Ton)	Energy Content (GJ/Ton)
Carbon Fiber	Conventional synthetic Fibers	12,500	130
Kevlar Fiber		75,000	25
Glass Fiber		1200–1800	30
Plant Fiber	Sustainable Alternation	200–1000	4

**Table 2:** Biochemical compositions of plant fibers with respect to their species and origins.

Source of Fiber	Species	Fiber Origin	Cellulose (%)	Hemicellulose (%)	Lignin (%)	References
Bamboo	(>1250 species)	Culm	40–55	18–20.8	15–32.2	[23,24,27,28]
Jute	*Corchorus capsularis*	Stem	56–71	29–35	11–14	[16,22,29]
Coir	*Cocos nucifera*	Fruit	32–43	21	40–45	[16,30,31]
Palm	*Elaeis guineensis*	Fruit	32–35.8	24.1–28.1	26.5–28.9	[32]
Sugarcane Bagasse	*Saccharum officinarum*	Stem	32–44	25	19–24	[22]
Water hyacinth	*Eichhornia crassipes*	Stem	43.58–47.38	19.77–22.23	9.52–13.08	[2,16,26]
Rice	*Oryza sativa*	Husk	59.9		20.6	[33]
Sisal	*Agave Sisiana*	Leaf	57–71	16	11–12	[28,34]
Flax	*Linum usitatissimum*	Stem	62–72	18.6–20.6	2–5	[2,22]
Banana	*Musa indica*	Leaf	60–65	25	5–10	[22,35]
Hemp	*Cannabis sativa*	Stem	67–78.3	5.5–16.1	2.9–3.7	[36,37]
Kenaf	*Hibiscus cannabinus*	Stem	70	3	19	[35]
Pine	*Pinus lambertiana*	Straw	67.29		11.57	[20]
Barely	*Hordeum vulgare*	Straw	33–40	20–35	8–17	[38]
Wheat	*Triticum aestivum*	Straw	30	50	15	[38]

**Table 3:** Physical and Mechanical Properties of potential plant fibers in reinforcing the soil.

Fiber	Density (kg/m^3^)	Young’s Modulus (GPa)	Ultimate Tensile Strength (MPa)	Elongation at Break (%)	Moisture Absorption (%)	References
Bamboo	715–1225	33–40	400–1000	-	40–52.45	[15,28,39,40,41]
Jute	1300–1450	10–30	393–860	1.5–1.8	12	[2,15,22,37]
Coir	1390–1520	3–6	100–225	12–51.4	130–180	[2,15,42]
Palm	463	26–32	100–400	19	1–10	[2,15,43]
Sugarcane Bagasse	1250	15–19	66.29–290	1.1	-	[22,44,45,46]
Water hyacinth	800	-	295.5–329.5	13.6	32	[2,16]
Rice Husk	-	-	-	-	-	-
Sisal	700–1330	9–20	400–700	3.64–13	56–230	[2]
Flax	1500	27.6–80	345–1500	1.2–2.7	7	[2,37]
Banana	1350	27–32	711–779	2.5–3.7	-	[22,45,47]
Hemp	1140–1470	30–70	690–920	16	8–9	[2,25,36,37]
Kenaf	1040	136	1000	-	307	[35]
Pine	813	-	61.65	10.68	-	[20,48]
Barley	870	-	-	-	400	[35]
Wheat	868	-	-	-	280–350	[35]

**Table 4:** Comparison of specific properties between conventional synthetic fibers and plant fibers.

Fiber Material	Fiber Type	Density (kg/m^3^)	Young’s Modulus (GPa)	Ultimate Tensile Strength (MPa)	Reference
Carbon Fiber	Conventional synthetic Fibers	1800	130	1710	[22]
Kevlar Fiber		1400	90	2710	
Glass Fiber		2600	30	1350	
Plant Fiber	Natural Fiber	Up to 1500	Up to 130	Up to 1500	Refer Table 3

**Table 5:** Summary of recommended treatments to natural fibers prior to soil reinforcing.

Fiber	Recommended Prior Treatments	Prime Targets	References
Bamboo	Heat treatment (in Oil at 150 °C for 4 h)	Enhance thermal stability, weather resistance	[40,57,69]
	Coating of bitumen or water-based paints	Prevention of water ingress, prevention of microbial degradation	
	Application of two-component epoxy resin (Enamel, ExaPhen)	Enhancement of composite bonding	
	Hot press	Densifying, strengthening	
Jute	4 h of alkali treatment in 5% NaOH and reinforcement using vinylester resin matrix at 30 °C	Increase surface roughness of fiber	[12,17,88,119,120,121]
	Reinforcement with polyester resin and Nano-clay	Increase strength, decrease water absorption	
	Coatings of bitumen or antimicrobial benzothiazole chemicals	Prevention of water ingress, prevention of microbial degradation	
Coir	Treatment with H_2_O_2_	Increase thermal stability, removal of waxes and fatty acids	[100,103,122,123]
	Treatment with phenol and bitumen	Enhancement of durability	
	Treatment using NaOCl/NaOH for the exposition of cellulose and hemicellulose	Reduction of water absorption	
	Treatment using CCl_4_	Prevention of microbial degradation	
Palm	Coating with acrylic butadiene styrene (ABS) thermoplastic to protect from biodegradation and to increase the friction with soil particles	Prevention of biodegradation, increase surface friction	[52]
Sisal	Acetylation by acetic anhydride	Modification of fiber cell wall as hydrophobic, increase rigidity and roughness of fiber	[2]
	Permanganate treatment	Reduce hydrophilic tendency	
Flax, hemp	Ultrasonic impact can be applicable	Increase durability	[2]
